# The Dental Situation Today and How to Meet It*Extracts from article entitled “Oral Hygiene in Relation to Public Health,” published in the *Journal of the National Dental Association*, April, 1920.

**Published:** 1920-06

**Authors:** Alfred C. Fones


### THE DENTAL SITUATION TODAY AND HOW TO MEET IT*

BY ALFRED C. FONES, D.D.S.

Dental caries is so exceedingly common that it is difficult to find two school children out of a hundred with perfectly sound teeth, and even in early childhood our school children average seven cavities per child. Consequently there are few young people who escape a pulp involvement due to the penetration of the bacteria through the tooth structure, destroying this delicate and sensitive tissue. Pulpless teeth are therefore, so prevalent that it is the exception for an adult over thirty years of age to present a mouth without one or more devital teeth which already have, or which may develop an apical infection.

This brings us to a realization that dentistry can no longer be considered as a luxury, but that dentistry in some form is an absolute necessity for every one. We are forced to the conclusion that we are now facing our most serious public health problem in these pernicious mouth conditions, and that there is no one factor so important to public health. Mouth hygiene must be considered as the most important and most pressing health measure.

With whom does the responsibility for the solution of this problem rest? The dental profession? If so, can the dental profession, as it exists today, cope with the problem? Let us make a brief analysis of the situation.

There are, in the United States, about one hundred and ten millions of people. From statistics based upon the examination of the teeth of children in the public schools in various parts of our country, we deduce that fully 95% of this number are afflicted with decayed teeth. There are, approximately, forty-eight thousand (48,000) dentists in

* Extracts from article entitled "Oral Hygiene in Relation to Public Health," published in the *Journal of the National Dental Association*, April, 1920.

the United States. These men can not give proper dental service to more than fifteen to twenty millions. There are in other words, but fifteen to twenty millions of people who are sufficiently educated to a realization of the importance of the care of their teeth, to visit the dentist with any degree of regularity. Even under these conditions the time of the average dentist is wholly filled with the relief, restorative and repair work needed by these people. This leaves eighty millions who give little or no attention to their mouths, excepting possibly to have a tooth out when it aches. Good dental service is expensive, so expensive, in fact, that it has always been considered a luxury. Must we not conclude, then, that the eighty millions of people for whom there is no provision in dentistry, form the great working classes to whom sound teeth are a necessity for good health?

Do we need more dentists? Yes. But how are we to secure them? The modern tendency of our dental colleges is to provide the profession with better equipped men, to raise the standards of entrance requirements, lengthen the college course and enlarge the curriculum, until eventually the average dental graduate will be twenty-five or twenty-six years of age. In a short time, the standards for dental education will equal those for medicine, and both the dental and medical students will be required to pass an academic college course before they enter the four years' professional course. And this is as it should be. The man who is to handle the serious conditions which are present in the average mouth must be fully equipped for this service, for to many of his patients it will be a matter of life or death.

From the foregoing analysis we are confronted with the fact that, although this public health problem should belong to the dental profession, the profession as it stands today is not able to shoulder it, and our colleges can never turn out graduate dentists fast enough to even cope with it. And we are further forced to admit that not only is the dental profession unable to cope with mouth hygiene as a public health measure, but the average dentist can not cope with the need for mouth hygiene as a private health measure, even for the patients in his own practice. For this condition we do not hold to account the dentist who admits that he can not cope with the appalling conditions alone, he has been pushed to the last notch in the attempt to meet the great flood of relief, repair and restorative work which has come upon him. But what of the dentist who opposes every effort to provide the dental profession with the help it must have and the public with the service it so badly needs?

We have known for many years that these pernicious mouth conditions are unnecessary. It has been proven that 80 to 90 per cent. of dental decay can be prevented by a system of extreme cleanliness and correct diet, especially in the elimination, or at least the restriction of the consumption of free sugar.

We know: (1) That the bacterial placque is the initial stage of dental caries, and that frequent removal of these plaques from all the surfaces of all the teeth by hand polishers is the most sufficient means, aside from a correct diet, for the prevention of dental caries. (2) That the frequent removal of all calcareous deposits around the necks of the teeth, by the use of instruments, is most effective in the prevention of infection and destruction of the dental tissues surrounding the roots of the teeth. (3) That the faithful use, daily, of the tooth brush and floss silk, and of a mouth wash, such as lime water made from coarse calcium oxide, is the best means for the thorough removal of food debris and dissolving the placques. (4) That nearly all micro-organisms in the human mouth are harmless, if deprived of a pabulum, such as food debris, upon which to feed, develop and multiply.

Now, if these are facts, how shall we make a practical application of them to aid the populace in preventing dental caries, pericemental infections, and in appreciating the importance of clean mouths and sound teeth?

The answer, in my judgment, is by means of the dental hygienist, a woman educated and trained in this specialty. The first effort to demonstrate the great service that such women could render was made in Bridgeport, Connecticut, in 1914, when the first class of dental hygienists was graduated. In 1915 a second, and in 1917 a third class was graduated. The efficiency of these women in carrying out educational and preventive measures, and the great benefits that have emanated from her services, has resulted in the establishment of organized training schools for dental hygienists at Columbia University, New York City; the Forsyth Dental Infirmary, of Boston; the Rochester Dental Dispensary, the University of Minnesota College of Dentistry, Minneapolis, and the Colorado College of Dental Surgery at Denver. The organization of several additional training schools is now under way, and during the past five years twelve states have amended their dental laws to permit of the practice of dental hygienists under the general supervision of the dentist. The dental hygienist is firmly established, the present demand for her services is far greater than the supply, and nothing can stop this educational and preventive movement.

The present need of the dental profession, in solving the public health problem of mouth hygiene, is an immense corps of women workers, educated and trained as dental hygienists and therefore competent to enter dental offices, infirmaries, public clinics, sanitariums, factories and other private corporations, to care for the mouths of the millions of adults who need this educational service so badly. The need in every state is so great that every state must provide its own training schools, and if the dental profession will not meet the situation, the state health or educational authorities must do it.

### JUVENILE DENTISTRY

The real field for prevention is not so much in the adult mouth, but is at the very source where the evil originates, and that is in the child's mouth. The greatest work to be done is in the public schools. For five years we have been demonstrating in the Bridgeport schools the value of an educational and preventive dental clinic, and it has developed into one of the most important parts of our school and health system.

Under the plan of this clinic, every child in the first five grades undergoes an examination of his mouth, and receives a prophylactic treatment of the teeth at regular intervals, accepting it as much a part of the school curriculum as his lessons. Every child in these grades is taught a method of brushing his teeth and is educated in the care of his mouth. In this way the municipality accepts one-half of the responsibility, that of educating and aiding the children in the prevention of dental decay, while the home care of the mouth and proper feeding must be assumed by the child and his parents—a plan on the fifty-fifty basis.

During the past year we have cared for the mouths of twenty thousand individual children with a corps of twenty-six dental hygienists, two supervisors and an assistant supervisor, who are also dental hygienists, and three women dentists. The service of the dental hygienist consists in the actual cleaning and polishing of the children's teeth in the schools, the examination and charting of the mouth conditions for permanent records, individual instruction in the care of the mouth, and toothbrush drills and talks in the classrooms. Supplementary to this are stereopticon lectures given by the supervisors and dentists for the education of children in grades four to eight. Illustrated pamphlets are sent to the home for the education of the parent and to gain co-operation.

This has been a pioneer work and was begun during the war period which produced very unsettled conditions in our schools. However, we have proven that the teeth of children carried through this five-year demonstration show, at the end of the period, a reduction of 33.9 per cent. in dental caries. This figure represents the average reduction of dental caries in the fifth grade of thirty schools. The record in some schools was quite remarkable, one school having as high as 67 per cent. and several with averages of over 57 per cent. The comparisons which we have been able to make in our health records also convince us that mouth hygiene is a very powerful factor in the reduction of communicable and infectious diseases in childhood.

We do not claim that mouth hygiene is a panacea for all the ills of the school child. Instead we are daily confronted with the fact that there is a crying need for a general health program of considerable magnitude for the prevention and correction of other remediable physical defects besides those found in the mouth.

### A NEW PROBLEM IN MEDICAL HYGIENE

Watching the children in the chairs of the hygienists in the schools, and noting some minor physical defects which are apparent to even a casual observer, I suddenly realized that the medical profession was confronted with a problem similar to that of dentistry.

With the long and expensive education and training now required for the prospective physician, could the medical profession hope to send medical men into the public school system in sufficient numbers to examine every child and record the condition of his eyes, ears, nose, throat, chest, back, feet, height and weight? Will they not also be obliged to have the data secured and the classifications made for them so that the trained medical mind may concentrate upon the final diagnosis? It hardly seems practical to attempt to add the physical examinations to the field of the medical nurse. Her training is such that she is indispensable in the care of those who are already sick.

For a moment, I would ask you to consider that, by training, neither the medical nurse nor the dental hygienist is especially fitted to undertake the physical supervision of the child, either working separately or together.

This brings us to a plan I would like to present to you to provide the children of our public schools with the same careful supervision of the physical development that is now accorded to the mental development. And just as the supervision and training of the child's mind is intrusted to one person, the teacher, so should one person be charged with the supervision of his body. Let us call this person the Public School Hygienist, a woman trained and educated dentally and medically to a sufficient degree to act as a physical inspector, prophylactic operator and teacher of health and hygiene. In other words, we will Burbank the medical nurse and the dental hygienist, and thereby eliminate the unavoidable friction of two workers with separate interests.

The public school hygienist will be supplied in much the same way as the public school teacher. She will be a high-school graduate trained in the state normal school for teachers, receiving, in addition to the regular normal school courses in pedagogics and psychology, the special medical and dental education to fit her for her field of service, and she will enter the public school system on the same basis as the public school teacher.

The practical application of this plan would be as follows: The public school hygienist would be permanently located in a school and charged with the supervision of a certain number of children. She would prepare a normal diagnosis for each child under her supervision. That would mean the recognition and recording of any deviations from the normal in the eyes, ears, teeth, nose, throat, chest, back, feet, height and weight.

The medical and dental men are thereby relieved of the necessity of expending an enormous amount of time and energy upon the sorting and classification of the normal child from the abnormal. The way is thus cleared for the concentration of the highly specialized mind upon the final diagnosis. The recommendations for the correction of remediable defects, either through the family physician and dentist, or through the municipal clinics. are made, and it becomes the responsibility of the school hygienist to work through the child and the home to the end that the defects are corrected.

Once the physical defects which interfere with proper growth and development are recognized and recorded, the school hygienist begins at once the teaching of health habits, the group competitions based upon weight and height, the cleaning and polishing of the teeth, the tooth-brush drills, the food talks—in short, the physical education and supervision of the child.

SUGAR A CAUSE OF CARIES

Cane sugar has been used for hundreds of years by different nations, and they have invariably shown a susceptibility to dental decay. Free sugar is not a natural food and Nature never intended that it should be extracted from the cane and beet to be consumed in excessive quantities, as it is today.

When we consider that dental caries can only be produced from starch and sugar and that the starch must be reduced to dextrose before it can be converted into lactic acid, it is quite truthful to make the statement that all dental decay is produced by sugar. In the Bridgeport schools we are using this truism in the following jingle:

"Children, you should learn this truth;
Nothing but sugar decays a tooth."

Clinical experience and general observation, however, seem to show that the high consumption of starchy food, if unaccompanied by free sugar, does not result in dental decay. In fact, the evidence is all against the sugar. Among the peasant classes of Italy, Greece, the Balkan states, Germany, etc., where the diet consists mainly of coarse foods, vegetables and fruits, but where free sugar is a luxury and can not be indulged in, decayed teeth are the exception and not the rule. This is also true of the Esquimos, the African negroes, the American Indians, the Maoris of New Zealand, and many of the South Sea Islanders.

In the examination of the mouths of many hundreds of draftees, we noted especially the mouths of the Italians, many of whom had thirty-two perfect teeth without a cavity or filling, and yet these men had reached twenty-one years of age without even owning a toothbrush, and had consumed quantities of starchy foods. They stated that they did not care for sweetened foods, and we found later that the free sugar consumption in Italy averages but thirteen pounds per capita per year, less than a teaspoonful a day. In this country we average nearly one hundred pounds per capita per year. The American mother would be inclined to question this average, but few realize the enormous amount used weekly in the average home for cooking alone.

The medical profession is, to a great degree, responsible for this situation, for the family physician has taught mothers to believe that free sugar is an essential food for growing children.

Under existing conditions, what chance has an American child to have sound teeth? If he is a modified milk baby, sugar is added to the milk in the proportion of one ounce in twenty, at only a few weeks after birth, and all too frequently cane sugar takes the place of milk sugar. The taste and craving for sweetened foods is developed at once and is steadily encouraged as he progresses to cereals with sugar, puddings, jellies, sweetened crackers, etc. To the normal sugar supply found in milk, vegetables, fruits, and in the conversion of starchy foods, is added an ever-increasing amount of free sugar at meal time, augmented between meals by soda water, ice cream and candy. The sugar consumption is so excessive that the liver is overloaded with glycogen, and I believe that herein lies the secret of the child's susceptibility—not only in the fermentation of the sugar on the teeth, but also in the action of osmotic forces through the enamel with the blood and body juices which are surcharged with glucose and the absorbed products of fermented surplus glucose from the intestines.

There can be but one result: the deciduous teeth are attacked by dental caries, and at the beginning of his school life the child presents a wrecked mouth and it is only a matter of time before the permanent teeth are similarly affected.

And so we find that the medical profession, by advocating free sugar as part of the diet, is constantly creating a disease known as dental caries, which demands a specialty known as dentistry. Dentistry, in turn, has filled, crowned and capped these decayed teeth in innocence and ignorance of the bacterial colonies which exist on the ends of the roots of pulpless teeth, causing secondary infections of the heart, kidneys, joints, etc., thus returning the compliment to the medical profession by creating thousands of cases of systemic infection to be given over to its care and treatment, with the public as the victim.

Neither the medical nor the dental profession has realized that this vicious circle existed, but no great reduction can be made in dental caries and resultant systemic infections until this circle is broken.

The American people have been slowly educated to a knowledge of the evils of alcohol until we now have national prohibition. Any great reform must be accomplished by the same painstaking enlightenment, and American mothers who have, for generations, been educated to look upon free sugar as a food, must now be taught that free sugar is the chief cause of dental decay and that dental decay is the chief cause of many of the serious illnesses of childhood and adult life.

A number of years ago one of the leading surgeons of the country made this statement: "The next great step in preventive medicine should be made by the dentists; the question is, will they do it?" I should like to amend that statement to have it read: "The next great step in preventive medicine should be made by the medical and dental professions, by advocating a correct diet for children from birth to fifteen years of age, and from which free sugar shall have been eliminated."

